# Chiral Molecules in Action: Chemistry of Chiral Perovskite and Perovskite-Inspired Materials

**DOI:** 10.1021/acsenergylett.5c02877

**Published:** 2025-10-24

**Authors:** Ramavath Babu, Julian E. Heger, Taniya Dutta, Xiaowen Hu, Narayan Pradhan, Peter Müller-Buschbaum, Sergio Gómez-Graña, Lakshminarayana Polavarapu

**Affiliations:** † CINBIO, Universidade de Vigo, Department of Physical Chemistry, Vigo, 36310, Spain; ‡ 84665 Technical University of Munich, TUM School of Natural Sciences, Department of Physics, Chair for Functional Materials, James-Franck-Str. 1, 85748 Garching, Germany; § School of Materials Sciences, 62397 Indian Association for the Cultivation of Science, Kolkata 700032, India; ∥ SCNU-TUE Joint Lab of Device Integrated Responsive Materials (DIRM), Guangdong Provincial Key Laboratory of Optical Information Materials and Technology, National Center for International Research on Green Optoelectronics, South China Academy of Advanced Optoelectronics, 12451 South China Normal University, Guangzhou, 510006, China

## Abstract

The emergence of chiral metal halides marks a pivotal advancement in materials science, where structural asymmetry enables unprecedented control over spin-selective transport and polarized light interactions for optoelectronic and spintronic technologies. The introduction of chiral ligands into the metal halide lattice or on the surface of NCs imparts chirality to the corresponding hybrid materials, which adapts the handedness (R or S) of the chiral molecule. The choice of chiral molecule and metal halide type critically influences the crystal structure and dimensionality of metal halide crystals and thus their properties. Despite significant progress, the relationship between structure and chiroptical efficiency remains unclear. Nonetheless, they show great promise for spin filtering, enabling the fabrication of chiral LEDs and photodetectors. Considering these advancements, this Perspective focuses on the chiral-ligand-assisted design, synthesis, and functional exploration of chiral metal halide bulk and nanocrystals, along with the outstanding challenges that need to be addressed in the future.

## Graphical abstract

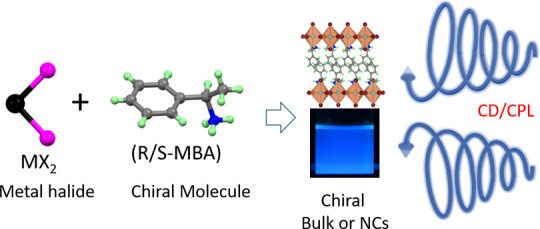

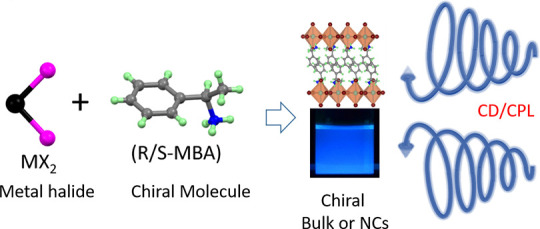

Organic–inorganic hybrid perovskites (OIHPs) and their derivatives have emerged as highly promising materials for strong light absorption, long carrier diffusion lengths, tunable bandgaps, and low trap-state densities,^1–4^ which have facilitated rapid progress in applications such as photovoltaics, light-emitting diodes (LEDs), and photodetectors, achieving impressive device performance with facile fabrication.^5–7^ Beyond these advantages, the structural flexibility of perovskites enables the incorporation of diverse organic cations, including chiral ligands, into their frameworks.^8–13^ Chirality, the property of asymmetry in which a system is non-superimposable on its mirror image, is widely observed in nature, from molecular structures such as DNA and peptides to macroscopic forms like seashells and flowers.^14,15^ Incorporation of such chiral molecules induces asymmetry in the lattice, either through structural distortion or the formation of chiral self-assembled architectures, giving rise to intrinsic chiroptical properties such as circular dichroism (CD),^10,12,16^ circularly polarized luminescence (CPL),^17,18^ and spin-selective charge transport.^12,16^ The chiral molecules in the lattice or on the surface of metal halides render them chiroptically active, adopting the handedness of the incorporated chiral molecules.

The chirality of molecules is harnessed in perovskite and perovskite-inspired crystals to induce chiroptical activity, with the handedness of the chiral molecules dictating the handedness of the resulting chiral crystals.

These features position chiral perovskites as promising candidates for spin-controlled optoelectronics, such as spin-polarized LEDs,^17,19^ spin generators,^20^ spin valves,^20^ spin solar cells,^21^ circularly polarized light detection,^22,23^ nonlinear optics,^10,24,25^ and ferroelectricity.^26,27^ The early reports on the synthesis of chiral OIHPs date back to 2003 ^28^ and 2006 ^29^ involving 1D and 2D single crystals, respectively. However, the field re-emerged in 2017 ^30^ with the observation of an interesting chiroptical response from hybrid 2D perovskite thin films, and subsequent studies have continuously revealed prominent chiral signatures and extended exploration in ferroelectrics,^31,32^ circularly polarized photodetectors,^22,33^ two-photon absorption-based upconverted circularly polarized luminescence,^34^ the Chiral-Induced Spin Selectivity (CISS) effect,^12,16^ self-driven X-ray photodetection^35^ and switching materials.^36^ In most cases, the chirality in metal halides is induced by chiral cations either incorporated into their lattice or bound to their surface. The choice of the chiral molecule and metal halide is critical for controlling the dimensionality of chiral metal halides. In the case of colloidal chiral NCs, the binding of chiral ligands to the surface or chiral template-assisted self-assembly induces chirality in the NCs.^37^ Both bulk and NC chiral metal halides have demonstrated strong potential for spin-controlled optoelectronics. Colloidal chiral perovskite nanocrystals serve as CPL-emissive media, while chiral low-dimensional perovskites act as spin filters in chiral LEDs. Recognizing the fast-growing field of chiral metal halides, this Perspective highlights the recent advances in synthetic methodologies that modulate chiroptical responses through chiral ligand selection and dimensionality control, which often crystallize in rare enantiomorphic space groups. Key properties, including CD, CPL, and spin-selective transport, are discussed in the context of their potential for next-generation spintronic and optoelectronic applications. Additionally, we discuss the synthesis of colloidal chiral perovskite NCs and their chiroptical properties, influenced by the selection of surface chiral ligands, which induce chirality in the NCs. Finally, we summarize the critical challenges that remain to improve chiroptical properties for future technologies.

Chirality in materials arises from the absence of mirror symmetry, often caused by asymmetrical atomic arrangements or bonding geometries. This concept is commonly illustrated by the “hand rule,” where left and right hands serve as a simple analogy for non-superimposable mirror images (see Figure 1a). The same principle applies to a wide range of molecules and crystalline structures in chemistry and materials science, where the lack of superimposability imparts unique physical and chemical properties. In such chiral materials, the lack of mirror symmetry enables selective interactions with circularly polarized light, in which the electric field rotates in a helical fashion, either left-handed (LCP) or right-handed (RCP), as shown in Figure 1b. Such systems can preferentially absorb one polarization over the other, leading to phenomena such as CD, where the difference in absorption between the LCP and RCP light provides a spectral signature of chirality. Upon excitation, they may also emit CPL. The typical preparation of chiral metal halides involves a chiral molecule, often in the form of ammonium halide, and a metal halide precursor. These two components crystallize in a solvent, resulting in chiral metal halide crystals, whose handedness (R or S) is determined by the chirality of the organic molecule, as shown in Figure 1c. The corresponding CD spectra of R and S-metal halides exhibit mirror-image-like CD spectra with opposite handedness. For example, R/S-methylbenzyl ammonium halide (MBA) and SnI_2_ crystallize into 2D (R/S-MBA)­2SnI_4_ perovskite as shown in Figure 1d. The chiral molecules are incorporated between the monolayer of corner-shared SnX_6_^4–^ octahedra, imparting chirality to the perovskite through asymmetric interactions. In the case of colloidal NCs, the chiral ligands bind to the surface, together with achiral ligands that stabilize the particles (Figure 1e). The surface chiral ligands impart chirality to the perovskite NCs. The dissymmetry factors (g_CD_ or g_lum_) of the chiral samples can be calculated by using the equations g_CD_ = *CD*(*mdeg*)/(32,980*A*), where CD is the signal intensity obtained from CD measurement and *A* is the absorbance, and g_lum_ = 2­(*I*_*L*_ – *I*_*R*_)/(*I*_*L*_ + *I*_*R*_), where *I*_*L*_ and *I*_*R*_ are the intensities of left- and right-CP light.

**Figure 1:**
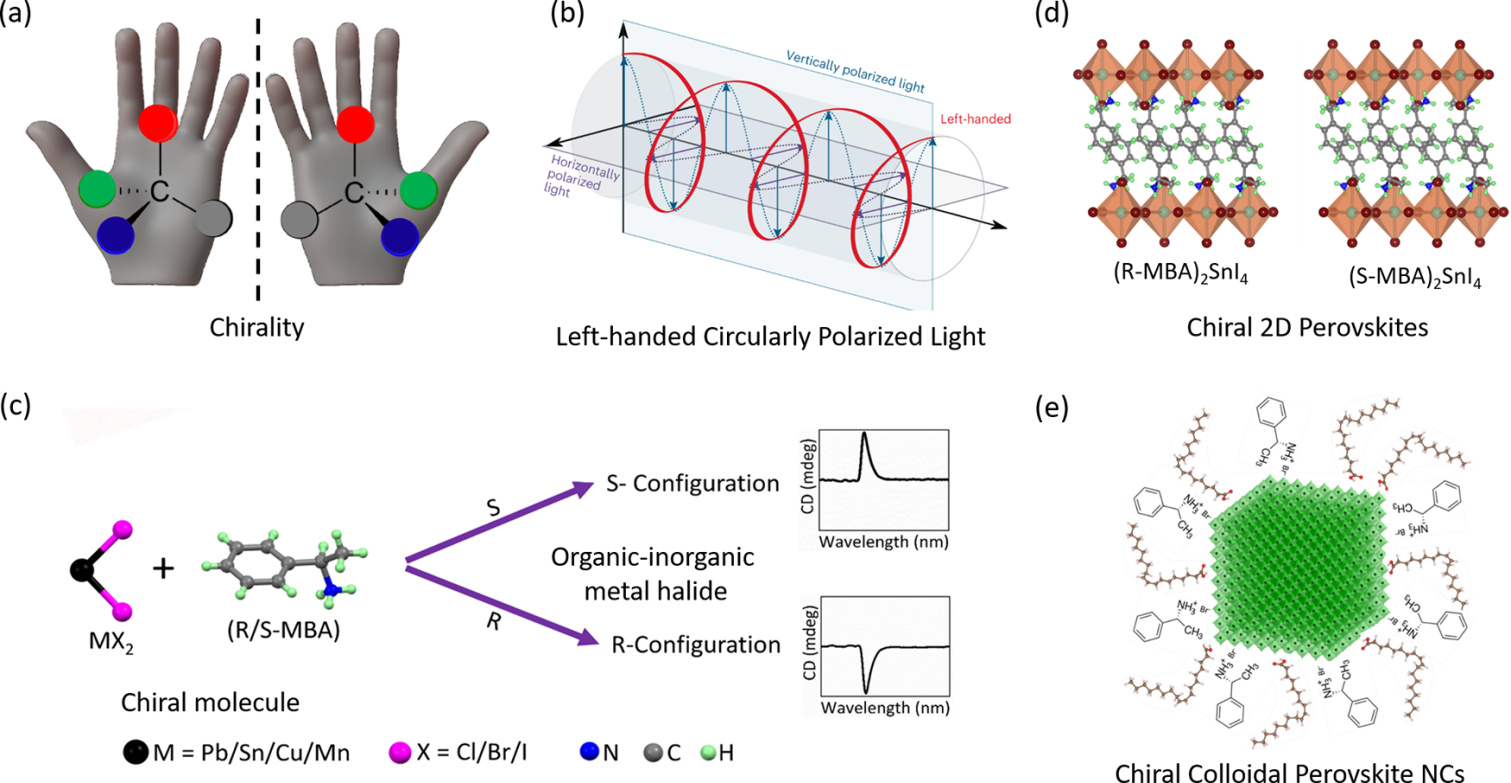
Overview of basic principles in chiral materials. (a) Chirality is a property of an object (e.g., hands) or material (amino acid molecules) that cannot be superposed on its mirror image by any combination of rotations and translations. (b) Circularly polarized light refers to the electromagnetic wave with a rotating (left or right, left is shown in the scheme) electric field vector perpendicular to its propagation. Reproduced from ref 38. Copyright 2024, Springer Nature. (c) Illustration of the synthesis of chiral metal halides via the reaction of metal halide precursors with enantiomeric chiral ligands (e.g., methylbenzyl ammonium halide). The resulting materials exhibit either S- or R-configurations depending on the type of chiral molecule, and they exhibit CD spectra with opposite signs. (d) The crystal structure of 2D chiral perovskites consists of metal halide octahedral monolayers separated by chiral organic molecules. (e) Schematic illustration of a chiral NC with a mixture of chiral and achiral ligands on its surface. In both cases, the chiral molecules induce the chirality, and the efficiency increases with decreasing dimensions of the bulk or NCs.

## Chiral Space Groups and Chiral Ligands

Chirality in crystals refers to the absence of improper symmetry elements such as mirror planes, inversion centers, and rotoinversion axes. As a result, only proper rotations and translations are allowed as symmetry operations in chiral space groups. Out of 230 available space group types, 165 possess at least one improper symmetry operation, such as inversion, mirror, glide, or Sn (a rotation combined with reflection in a plane perpendicular to the rotation axis). Therefore, crystals belonging to these space groups are always achiral, even if the three-dimensional asymmetric unit is chiral. In the context of chirality, only the 65 Sohncke space groups allow for chiral (handed) crystal structures, as shown in Figure 2a, arranged by crystal system, point group symmetry, and specific space groups,^39^ out of which 22 are enantiomorphic helical space groups (11 pairs) that include at least one screw axis, as shown in Figure 2a (highlighted in red).

According to crystallographic classification, 65 of the 230 space groups are Sohncke (chiral) groups, which include 11 enantiomorphic pairs that exhibit helical symmetry.

Crystals in these space groups are inherently chiral and exhibit helical enantiomorphic right- or left-handed structural chirality, even when the asymmetric unit itself is achiral, due to the presence of a chiral inorganic sublattice. The remaining 43 Sohncke space groups (highlighted in gray in Figure 2a) are characterized by the lack of mirror planes and inversion centers, so they can host chiral molecules in their lattice. However, they do not distinguish between left- and right-handedness, meaning they are not themselves chiral and can accommodate both chiral and achiral structures. In this class, each chiral space group has a mirror-image counterpart, meaning that R- and S-enantiomers of chiral metal halides share the same space group.^12^ Their non-centrosymmetric nature can give rise to macroscopic physical properties such as piezoelectricity, second-harmonic generation (SHG), and ferroelectricity. The crystallographic relationship between Sohncke space groups and the corresponding properties arising from the non-centrosymmetric structure can be described by the Neumann-Curie principle, as illustrated in Figure 2b.^39^ The structural and compositional diversity of chiral OIHPs provides considerable flexibility for tuning their optoelectronic properties, which is manifested in the multiple dimensionalities found in these materials, including three-dimensional (3D) networks, two-dimensional (2D) layers, one-dimensional (1D) chains, and zero-dimensional (0D) isolated structures. In these structures, chiral molecules act as A-site cations; therefore, they are used in the form of ammonium halides. The typical chiral molecules in the preparation of chiral metal halides are summarized in Figure 2c. Understanding the interplay between crystal symmetry and chiroptical properties provides a foundation for the rational design of OIHPs with targeted optoelectronic functionalities.

**Figure 2:**
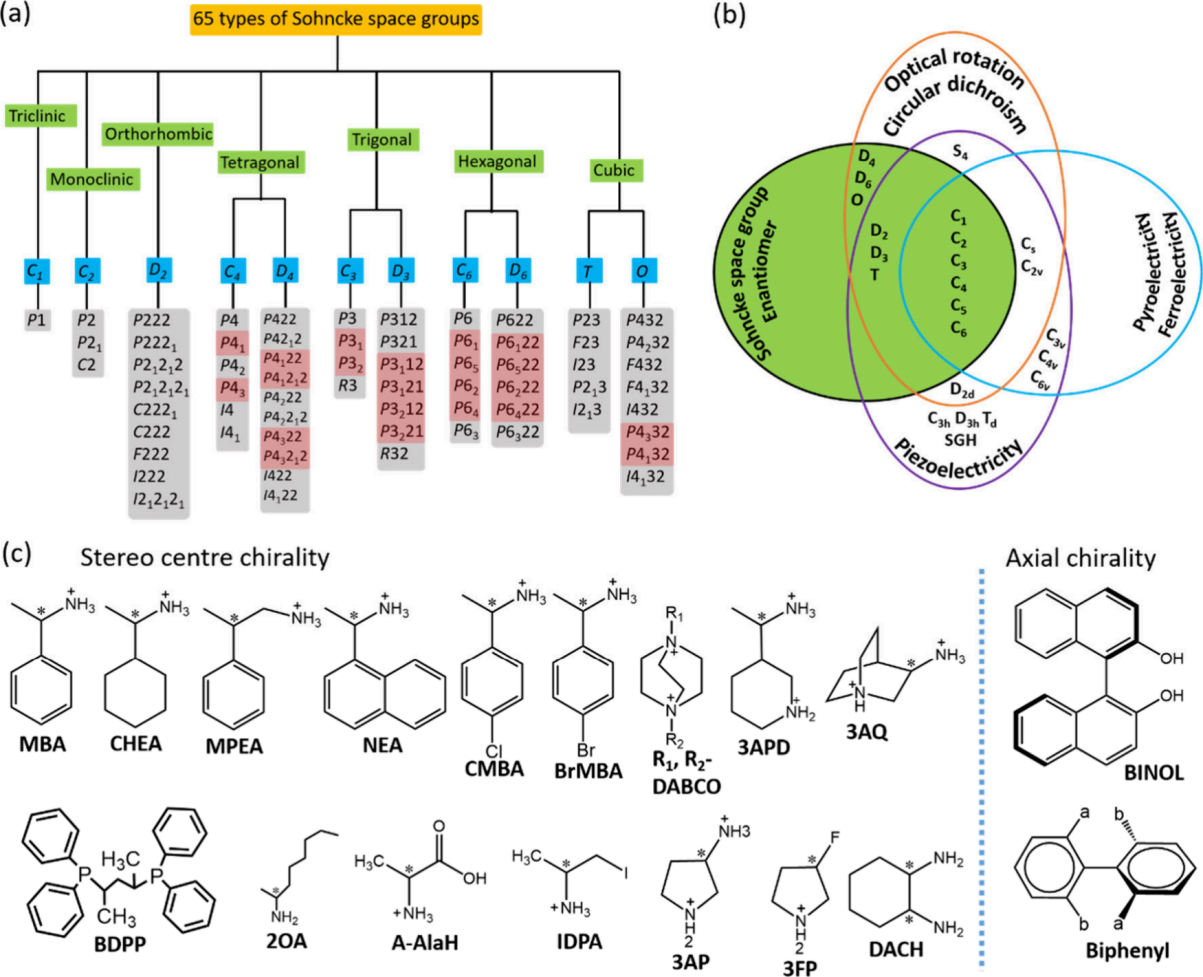
(a) The 65 Sohncke space groups can be organized according to their Bravais lattice and point group. The 22 helical chiral enantiomorphic space group pairs, which are intrinsically chiral, are highlighted in red. The remaining 43, shown in gray, are not intrinsically chiral, but they can still accommodate chiral molecules, resulting in chiral crystal structures. Importantly, any crystal structure belonging to one of these 65 Sohncke space groups is considered chiral, because these space groups lack symmetry operations that invert handedness, such as mirror planes, inversion centers, or rotoinversion axes. (b) The Neumann-Curie principle defines the relationship between crystal symmetry and the corresponding properties of non-centrosymmetric crystals. Figures a and b are inspired by ref 39. (c) A detailed schematic representation illustrating both stereocenters and axial chirality. **MBA:** methylbenzylammonium, **CHEA:** 1-cyclohexylethylammonium, **MPEA:** β-methylphenethylammonium, **NEA:** 1-(1-naphthyl)­ethylammonium, **CMBA:** 1-(4- chlorophenyl)­ethylammonium, **BrMBA:** 1-(4-bromophenyl)­ethylammonium, **R**_**1**_,**R**_**2**_ **-DABCO:** 1-alkyl-1,4-diazabicyclo-[2.2.2]­octan-1-ium, **3APD:** *R*-3- aminopiperidine, **2OA:** 2- octylamine, **3AQ:** 3- ammonioquinuclidinium, **BDPP:** 2,4-bis­(diphenylphosphino)­pentane, **A-AlaH:** protenated α-alanine, **IDPA:** 1-iodopropan-2-ammonium, **3AP:** 3- ammoniopyrrolidinium, **3FP:** 3- fluoropyrrolidinium, **DACH:** 1,2- diaminocyclohexane, **BINOL:** 1,1′-bi-2-naphthol.

## Synthesis Strategies for Chiral Metal Halide Single Crystals and Thin Films

Chiral perovskites and perovskite-inspired materials can be synthesized using two primary strategies: the *in situ* and *postsynthetic* approaches. The *in situ* approach provides a straightforward route to prepare chiral perovskites, where chiral organic ligands are directly introduced into a reaction mixture containing metal halide precursors, allowing for the spontaneous formation of chiral perovskite structures (single crystals or thin films) during the synthesis process (see Figure 1c).^12,16,40–42^ On the other hand, the *postsynthetic* approach involves chiral molecules that are subsequently attached to the surface of preformed perovskite structures, followed by surface functionalization to induce chirality in the resulting composites.^43,44^ In both processes, the induction and stabilization of chirality rely on noncovalent interactions, such as hydrogen bonding, electrostatic interactions, and π–π stacking. The *in situ* method is generally considered more efficient than the *postsynthetic* approach, as it effectively introduces chirality into the OIHPs. This enables the formation of structurally chiral materials with diverse dimensionalities, ranging from 0D molecular units to 1D chains, 2D layered structures, and 3D frameworks.^45^ Precise control over chirality across these structural dimensions is achieved through the rational selection of chiral organic ligands (Figure 2c) and metal halides or oxides. For instance, the combination of chiral MBA ligands with CuX_2_ (where X = Cl and Br) leads to the formation of 0D structures,^40,42^ whereas the use of MBA with SnI_2_ results in 2D layered structures.^12^ In this context, most chiral perovskites and perovskite-inspired materials are synthesized by dissolving precursors directly in concentrated hydrohalo acid solutions at elevated temperatures, followed by gradual cooling to room temperature, a process known as the temperature-lowering method (TLM).^13,46,47^ In addition, some of the chiral perovskites have been synthesized using an autoclave and antisolvent vapor-assisted (AVS) reactions. The autoclave reaction proceeds under high pressure and temperature within a sealed vessel to promote crystal formation,^48,49^ whereas the AVS method involves introducing a precursor solution to the vapor of a poor solvent (antisolvent), which slowly diffuses into the solution, reducing solubility and inducing controlled crystallization under mild conditions.^24^ The use of hydrohalic acid solutions is essential in both TLM and autoclave reactions, as they act as a halide source in the reaction medium. In contrast, the AVS method does not require hydrohalo acids, as halides are introduced directly in ionic form via the precursor solution. The choice of chiral ligands plays a crucial role not only in directing the dimensionality but also in modulating both the degree of chirality and the optoelectronic properties of the resulting hybrid materials. However, low-dimensional hybrid materials typically incorporate a higher proportion of chiral organic ligands and are therefore expected to exhibit an enhanced degree of chirality. For instance, in quasi-2D perovskites, a decrease in the anisotropy factor (*g*_abs_) was observed as the average number of inorganic layers between chiral organic ligands (*n*) increased.^50^ To date, only a limited set of chiral ligands has been employed in perovskite and perovskite-inspired systems (see Figure 2), likely due to challenges related to compatibility, stability, and effective incorporation into the perovskite lattice. Therefore, extensive exploration of chiral OIHPs synthesis using diverse chiral organic ligands is essential for advancing future optoelectronic and spintronic applications.

## Structure-Properties Relationship

As discussed above, chiral OIHPs exhibit diverse dimensionalities, ranging from 0D to 3D structures, depending on the spatial distribution of their organic and inorganic components, as illustrated in Figures 3, 4 and 5 and summarized in Table 1. These chiral OIHP structures commonly crystallize in the orthorhombic system with the chiral space group *P2*_*1*_ *2*_*1*_ *2*_*1*_.^12,40,42,45,51,52^ In exceptional cases, they crystallize in the tetragonal crystal system and enantiomorphic space groups such as *P4*_*1*_ *2*_*1*_ *2* or *P4*_*3*_ *2*_*1*_ *2*, which possess 4-fold symmetry^45,53,54^ Additionally, we have recently discovered 0D *(R-/S-MBA)*_*2*_ *SnBr*_*6*_ crystals that exhibit a hexagonal crystal structure with enantiomorphic *P6*_*1*_ and *P6*_*5*_ space groups (Figure 3a). Owing to this symmetry, the R- and S-enantiomers exhibit a distinct helical twist along one of the rotational axes (the *c*-axis), which stands in stark contrast to structures with 4-fold symmetry. Furthermore, the chiroptical properties and dissymmetry factors of our new materials are significantly enhanced by nearly 2 orders of magnitude compared to those reported for samples with 4-fold symmetry. As shown in Figure 2a, several enantiomorphic space groups have yet to be explored, offering unique opportunities in this research line. Therefore, broader exploration and targeted design of these space groups are required to advance chiral optoelectronic and spintronic applications.

**Figure 3:**
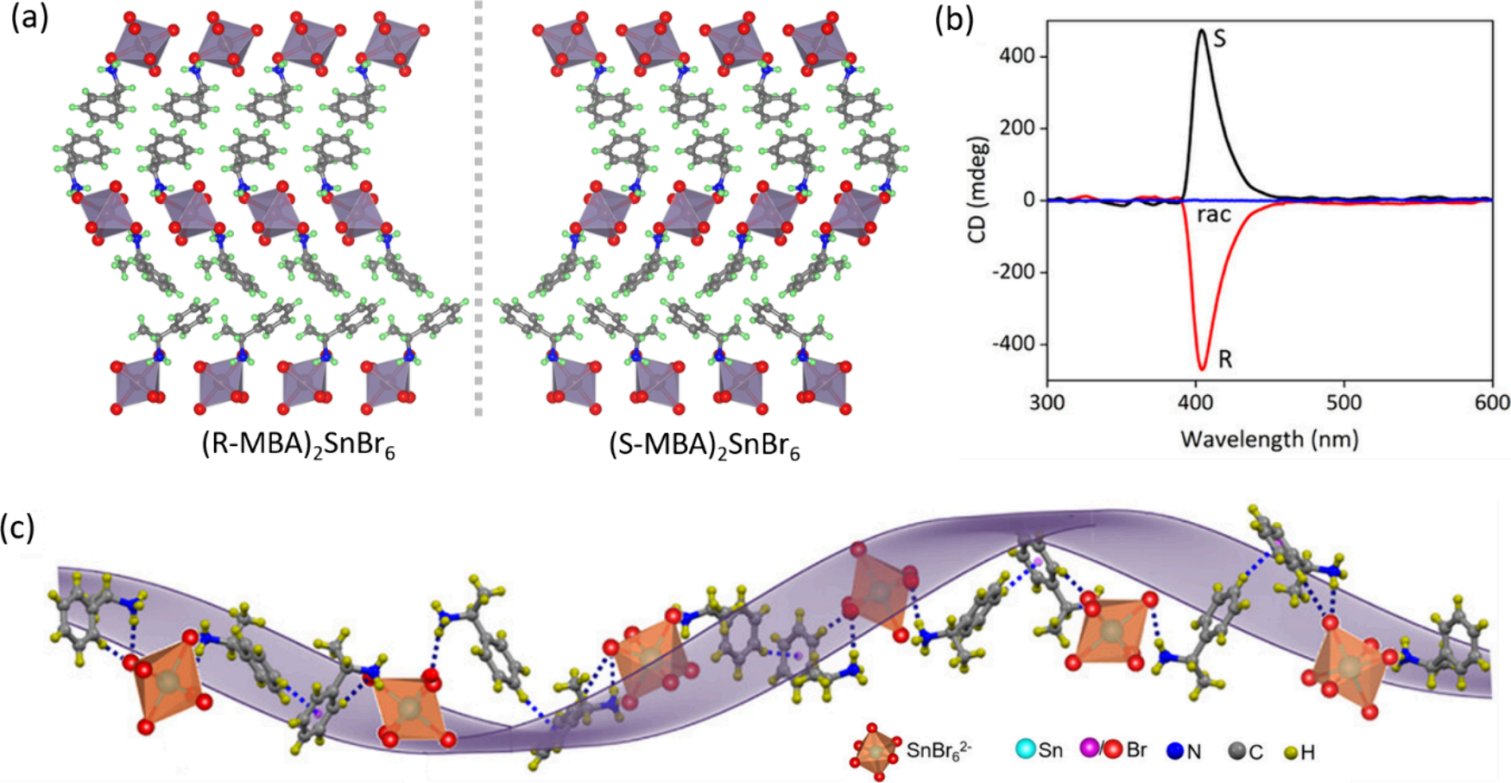
(a) Crystal structures of chiral (R-MBA)_2_SnBr_6_ and (S-MBA)_2_SnBr_6_ crystals viewed along the c-direction. (b) CD spectra of the corresponding crystals together with the racemic crystal. Crystals with opposite handedness show mirror-image CD spectra with opposite signs, whereas the racemic crystal displays no CD response. The absorption and CD maxima overlap. (c) Ball-and-stick model of the crystal structure viewed along the 6-fold screw axis, illustrating the helicity of the framework, where chiral molecules guide the helical arrangement of SnBr_6_^2–^ octahedral units. The violet color helix is a guide to the eye. The figure is reproduced from ref 55. Copyright 2025 Wiley-VCH GmbH.

**Figure 4:**
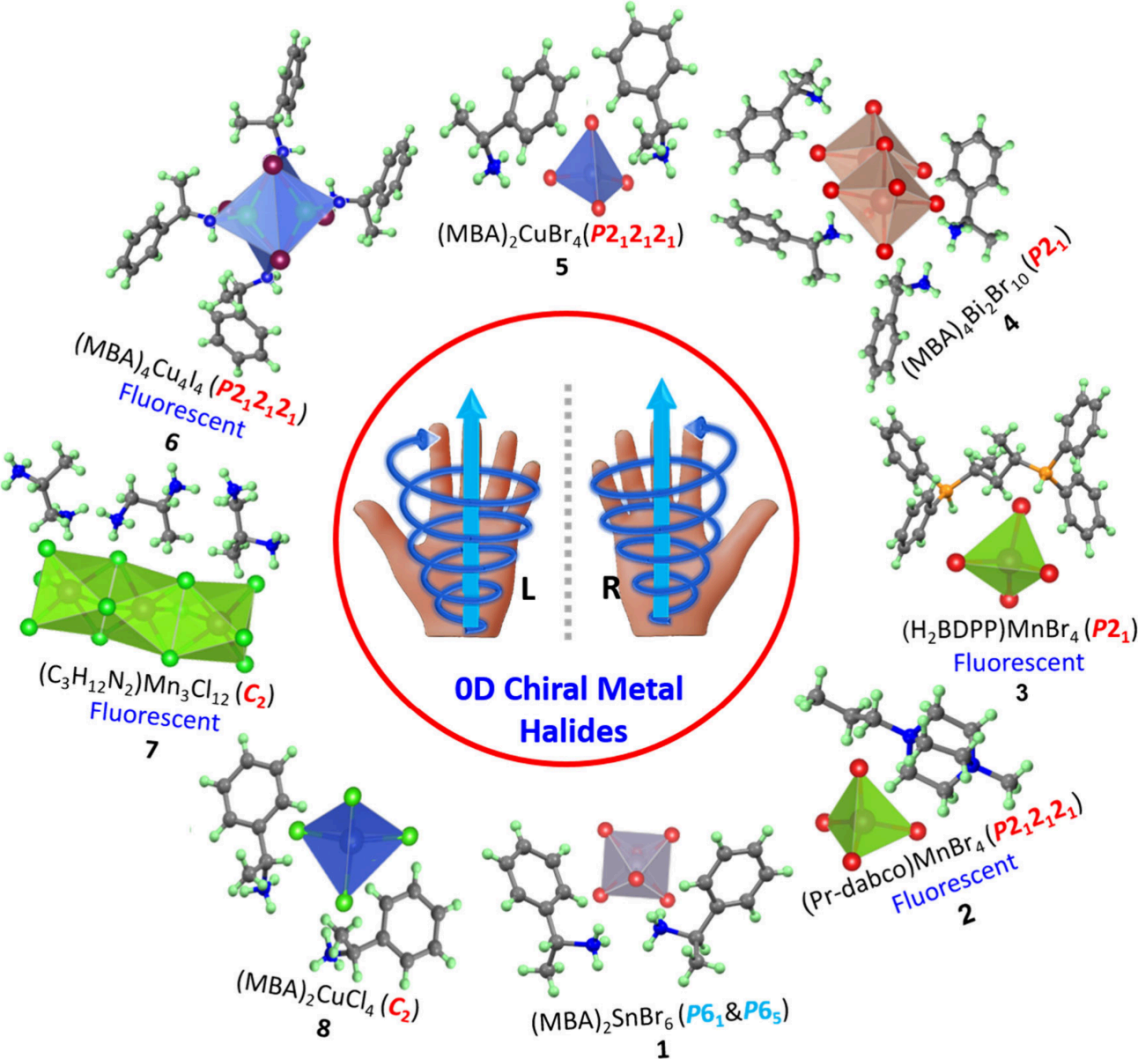
Asymmetric units of different chiral 0D metal halides reported in the literature are summarized. It illustrates the structural diversity arising from different metal halide frameworks and chiral organic ligands. These structures feature isolated metal halide polyhedra stabilized by chiral cations, forming zero-dimensional assemblies. The distinct arrangements within the asymmetric units highlight how chirality and local coordination environments influence the overall crystal packing and symmetry. The copper and manganese halide-based 0D chiral crystals exhibit CPL. The related works can be found in these references: 1),^55^ 2),^56^ 3),^58^ 4),^65^ 5),^40^ 6),^51^ 7),^59^ 8)^40^

**Figure 5:**
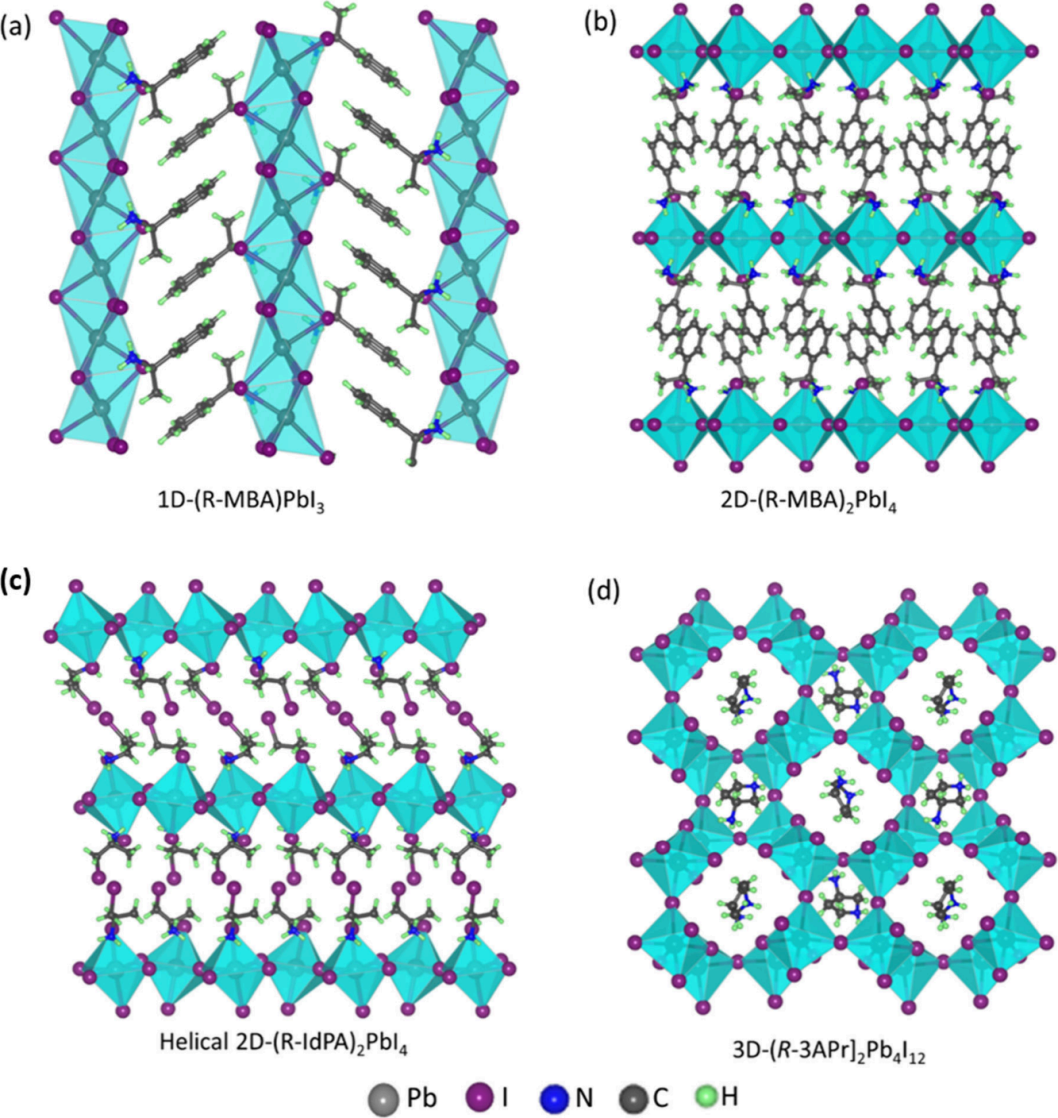
Chiral crystal structures of perovskites with different dimensionalities. (a) One-dimensional chiral perovskite, where the corner-sharing or edge-sharing octahedra extend linearly along a single crystallographic axis. (b) Two-dimensional chiral layered perovskites consist of inorganic sheets of corner-connected octahedra separated by chiral organic spacer cations. (c) Helical two-dimensional perovskites, where in-plane connectivity exhibits screw-like, helical distortions due to asymmetric coordination environments. (d) Three-dimensional chiral perovskites are characterized by a continuous network of corner-sharing octahedra in all three spatial dimensions. Related structures are adapted from the literature as follows: (a) ref 22, (b) ref 30, (c) ref 53, and (d) ref 49.

**Table 1:** Different Chiral Metal Halide Bulk Systems Reported in the Literature and Their Crystal Space Groups, Along with Their Dimensionality and CD and CPL Peak Maxima

Formula	Space group	Dimension	CD (nm)	CPL (nm)	Ref
(R/S-MBA)_4_Cu_4_I_4_	*P2*_1_2_1_2_1_	0D	±310	±630	^51^
(R/S-CTA)_2_CuCl_4_	*P3*_1_2_1_ *and P3*_2_2_1_	0D	–415, 380, 323, −294	--	^61^
(*R/S-*MBA)_2_PdCl_4_	*C*_2_	0D	±350	--	^41^
(R/S-MBA)_2_CuCl_4_	*C*_2_	0D	±378, ± 266, ± 226	--	^23,40,75^
(R/S-MPEA)_2_SnBr_6_	*P2*_1_	0D	±352, ± 268		^76^
(*R*/*S*-MBA)_2_CuBr_4_	*P2*_1_2_1_2_1_	0D	±290, ± 250	--	^40^
(R/S-MBA)_2_CuBr_4_	*P2*_1_2_1_2_1_	0D	±710–325	--	^42^
(R/S-MBA)_2_SnBr_6_	*P6*_1_ *and P6*_5_	0D	±390	--	
(R/S-DABCO)MnBr_4_	*P*2_1_2_1_2_1_	0D	±(200–400)	–541, 547	^56^
[H_2_(*RR*/*SS-*BDPP)] MnBr_4_	*P2*_1_	0D	±(300 to 500)	±530	^58^
(R/S–C_3_H_12_N_2_)Mn_3_Cl_12_	*C*_2_	0D	±(300 to 550)	±630	^59^
(S-MBA)_2_PbBr_3_	*P2*_1_2_1_2_1_	1D	--	--	^68^
(R/S-MBA)_2_PbBr_3_	*P2*_1_2_1_2_1_	1D	365		^69^
(R-APD)PbCl_4_·H_2_O	*P*2_1_2_1_2_1_	1D	320	--	^25^
(*R*/*S*-MBA)_2_PbI_3_	*P*2_1_2_1_2_1_	1D	±328, ±392	±395	^22^
(*R/S-*MBA)_2_PdBr_4_	*P*2_1_2_1_2_1_	1D	±450	--	^41^
(*R*/*S*-MBA)(GA)PbI_4_	*P*2_1_	1.5D	±340, ±370, ±420, ±510	--	^16^
(R-/S-MBA)_2_PbI_4_	*P*2_1_2_1_2_1_	2D	--	--	^29^
(R-/S-MBA)_2_PbI_4_	*P*2_1_2_1_2_1_	2D	±367, ±408, ±489, ±501	--	^20,30^
(R-/S-MBA)_2_PbI_4_	*P*2_1_2_1_2_1_	2D	±519	--	^70^
(R/S-MBA)_2_SnI_4_	*P*2_1_2_1_2_1_	2D	±357, ±402, ±443, ±473	--	^12^
(*R*/*S*-3-AQ)_2_PbBr_4_·2Br	*P*4_1_2_1_2 and *P*4_3_2_1_2	2D	±350	--	^45^
(R3HP)_2_RbBiBr_6_	*P*_1_	3D	--	--	^72^
(*R*/*S*-3AQ)Pb_2_Br_6_	*P*2_1_2_1_2_1_	3D	±330, ±350, ±370, ±378	--	^45^
(R/S-3Apr)_2_Pb_4_I_12_·2H_2_O	*P*2_1_	3D	±390, ±430	--	^49^

The optical properties of chiral OIHPs vary depending on the dimensionality of the structure, ranging from 3D bulk frameworks to 2D layered perovskites and even 0D molecular clusters.

In general, chiroptical activity increases as the dimensionality of chiral crystals decreases, progressing from 3D through 2D and 1D to ultimately 0D structures. However, further studies are needed to confirm this trend fully.

For instance, 3D (R-/S-3AQ)­Pb_2_Br_6_ (where 3AQ = 3-aminoquinuclidine) chiral perovskites exhibit mirrored CD spectra with multiple peaks between 320–380 nm, within a range of +4 to −6 millidegrees.^45^ Unlike the 3D structure, the 2D analogue (R-/S-3AQ)_2_PbBr_4_·2Br, which shares the same organic and inorganic components, displays a single mirrored CD peak at 350 nm with significantly higher intensity, ranging from +20 to −20 millidegrees, resulting in an approximately 1 order of magnitude increase in the dissymmetry factor.^45^ Therefore, the relatively weaker optical activity and narrower optical bandgap of the 3D structure, compared to its lower-dimensional counterparts, make it more suitable for photovoltaic applications. In contrast, 2D chiral perovskites exhibit enhanced spin–orbit coupling and higher exciton binding energies due to quantum confinement effects, resulting in stronger CD signals and making them highly promising for chiral optoelectronic and spintronic applications. Meanwhile, 1D and 0D chiral perovskites demonstrate even more pronounced chiroptical responses, attributed to their reduced dimensionality and localized electronic states.^40,41^ As a result, these systems display sharp excitonic features and intense CPL.

### 0D Chiral Metal Halides

The 0D OIHPs are a class of materials in which the metal halide units (inorganic polyhedral: e.g., [MX_6_]^4–^, where M = metal, X = halide) are isolated and surrounded by organic cations, which precludes any corner-, edge-, or face-sharing connectivity with neighboring units, as depicted in Figure 3a, with typical asymmetric units summarized in Figure 4. The handedness of the crystals can be controlled by the chiral molecule incorporated in the lattice. The R and S chiral crystals exhibit mirror-image like CD spectra with opposite signs, and their maxima overlap with the absorption maxima (Figure 3b). Importantly, the helicity of the inorganic lattice is guided by the chiral molecules incorporated in the lattice, and thus, the overall chirality is dictated by the handedness of the chiral molecule, as shown in Figure 3c. For example, in chiral (S/R-MBA)_2_SnBr_6_ crystals, the 60° rotation of the chiral molecules and the octahedral units with respect to their neighboring units leads to the formation of a helical crystal structure. The twist in the chiral molecules and their interactions with the inorganic units guide the overall crystal structure regardless of their dimensionality. The 0D OIHPs have predominantly been studied with transition metals, particularly copper (Cu) and manganese (Mn), typically adopting tetrahedral coordination geometries (CuX_4_ and MnX_4_, where x = Cl, Br, and I), as summarized in Table 1. Cu-based polyhedra are primarily stabilized by the MBA cation,^40^ whereas Mn-based polyhedra have been investigated with a broader range of organic ligands, including DABCO,^56^ MPEA,^57^ BDPP,^58^ 3AP,^59^ etc.^60^ As shown in Table 1, the majority of 0D chiral OIHPs crystallize in the orthorhombic chiral space group *P2*_1_2_1_2_1_, whereas only a few examples, specifically, one each of the R- and S-enantiomers, have been reported in the enantiomorphic trigonal space groups *P3*_1_2_1_ and *P3*_2_2_1_, respectively.^61^ Interestingly, Cu-based chiral 0D OIHPs exhibit strong CD with a record dissymmetry factor (*g*_a__b__s_) reaching up to 0.37 along with remarkable spin-dependent charge filtration as high as 90%,^40^ whereas Mn-based chiral 0D structures demonstrate efficient circularly polarized luminescence (CPL) with a maximum *g*_lum_ value of 0.007,^56^ highlighting their distinct potential for advanced chiroptoelectronic applications. By precisely regulating the stoichiometric ratio of R-/S-MPA chiral ligands and CdCl_2_ precursor, both 0D and 1D chiral OIHPs were synthesized, each featuring tetrahedrally coordinated [CdCl_4_]^2–^ units but with distinct structural dimensionalities.^62^ Interestingly, the 0D chiral perovskite exhibited ferroelectric behavior accompanied by CD, whereas the 1D structure, crystallizing in the polar ferroelectric space group *P1*, comprised four organic cations and corner-sharing [CdCl_4_]^2–^ tetrahedra forming chains along the *b* axis, resulting in pronounced CD, enhanced ferroelectricity, and an increased SHG response. Besides, a few studies have reported 0D Tin (Sn),^55^ Bi Bismuth (Bi),^63^ and Antimony (Sb)^64^ chiral metal halides with interesting chiroptical properties. It has been shown that air-stable Bi-based chiral metal halides, (R-/S-MBA)_4_Bi_2_Br_10_, are promising for spin filtering and can detect circularly polarized light.^65^ Notably, a high spin polarization of 80% was achieved, attributed to a large anisotropy factor of 0.3. As previously mentioned, the 0D (R-/S-MBA)_2_SnBr_6_ with a helical lattice exhibits broadband SHG, achieving a notable dissymmetry factor of up to 0.44. Importantly, some of these 0D chiral metal halides emit CPL with photoluminescence quantum yields, while the 2D hybrid perovskites are weakly luminescent. Especially, chiral Mn halides emit strong green photoluminescence that is attributed to the spin-forbidden d-d electronic transitions (^4^T_1_ → ^6^A_1_) of the Mn^2+^ ions and ligand-to-metal charge transfer (LMCT) transitions (Figure 4).^56,58^ In addition, some 0D Indium (In)-,^66^ Tin (Sn)-,^67^ and Antimony (Sb)-^64^ halides also emit intense CPL with *g*_lum_ in the range of 10^–2^-10^–3^. These 0D metal halides in achiral form exhibit luminescence primarily due to self-trapped exciton (STE) emissions. However, upon inclusion of chiral ligands, this emission becomes circularly polarized, opening up a wide range of applications.

### 1D Chiral Metal Halides

The 1D chiral OIHPs are composed of inorganic polyhedra (typically octahedra) connected along a single crystallographic axis via corner-, edge-, or face-sharing, resulting in extended one-dimensional chains, as shown in Figure 5a. These inorganic 1D chains are spatially separated by bulky organic cations, which occupy the interchain voids and serve to isolate the chains from one another. As a result, the overall structure exhibits strong anisotropy, with confined charge carrier dynamics and structural rigidity primarily along the chain direction (Figure 5a). By taking advantage of the unique structural characteristics, recent studies have introduced a range of novel 1D chiral OIHPs with remarkable chiroptical properties, opening new avenues for their application in next-generation optoelectronic technologies. Despite this progress, most reported chiral OIHPs and related compounds remain Pb-based and typically crystallize in the orthorhombic chiral space group *P2*_*1*_ *2*_*1*_ *2*_*1*_, as summarized in Table 1. One of the earliest examples of such materials was reported by Billing and Lemmerer in 2003 with the compound (S-MBA)­PbBr_3_, in which the octahedral units are connected through face-sharing to form a one-dimensional chain in the chiral MBA lattice.^68^ Later, enantiopure single crystals of (R-/S-MBA)­PbX_3_ (X = Br, I) and (R-/S-MBA)_2_PbI_4_ were prepared by controlling the precursor ratios, and their chiroptical properties, including nonlinear optical (NLO) activity and photoluminescence (PL), were investigated.^69^ Interestingly, (R-/S-MBA)­PbX_3_ exhibits a 1D structure that is CPL active with a PLQY of 1.5 and 1.4% for the R and S enantiomers, respectively, whereas (R-/S-MBA)_2_PbI_4_ adopts 2D structures and shows no such CPL activity. Furthermore, a class of chloride-based chiral 1D OIHPs was synthesized, in which highly distorted PbCl_6_^2–^ octahedra are connected via edge-sharing and stabilized within a chiral 3APD ligand framework.^25^ These structurally unique materials were further utilized in the fabrication of a white-light-emitting device, exhibiting exceptional white-light emission with a remarkable color rendering index (CRI) of 93.9. In addition, iodide-substituted (R-/S-MBA)­PbI_3_ enantiomers were synthesized and evaluated for their chiroptical properties, revealing an anisotropy factor (g_CD_) of approximately 0.02.^22^ Owing to their strong anisotropy factor, these materials were further used in circularly polarized light (CPL) detection, resulting in a device with a responsivity of 797 mA W^–1^, detectivity of 7.1 × 10^11^ Jones, a 3-dB bandwidth of 150 Hz, and stable performance sustained over one month (Figure 6). These results highlight that the combination of light-sensitive CP absorption from chiral organics and efficient charge transport in the inorganic framework enables high-performance CPL detection without additional optical elements. The solution-processable nature of these materials also allows fabrication of flexible devices on substrates such as polyethylene terephthalate, with performance comparable to rigid devices, demonstrating their potential for integrated, wearable, and flexible photonic applications. More recently, Pd-based 1D square-pyramidal coordination geometry and corner-sharing connectivity were reported, featuring non-centrosymmetric arrangements driven by halide (Cl and Br) interactions in the MBA organic matrix.^41^ These chloride, bromide, and mixed halide variants of 1D square-pyramidal chains exhibited exceptionally high structural distortion values in the range of 0.127–0.128. Due to their pronounced structural dissymmetry, these chiral OIHPs were further utilized in spin-filtering studies, demonstrating their potential for future spintronic applications. Additionally, representative crystal structures, including their space groups and chiroptical properties such as CD and CPL peak positions, are summarized in Table 1. Overall, these findings demonstrate the structural robustness, chemical tunability, and multifunctional potential of 1D chiral OIHPs, underscoring their promise for advanced optoelectronic and spintronic applications.

**Figure 6:**
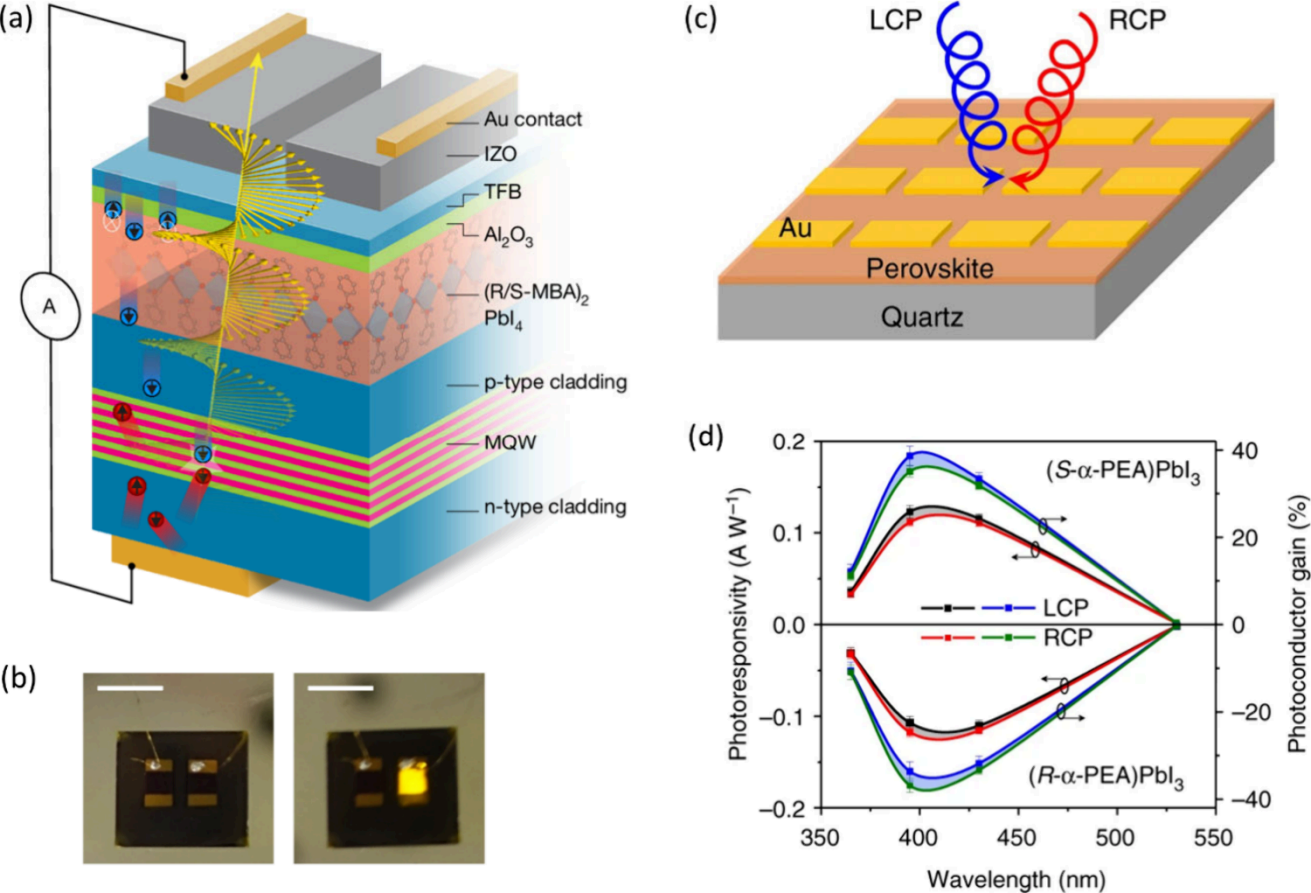
Device fabrication and functionality of chiral perovskites in spin-optoelectronic devices. (a) Stack of the spin-LED emitting circularly polarized electroluminescence (CP-EL). The (R-MBA)_2_PbI_4_ acts as a spin filter, allowing only spin-polarized holes (blue circles) to flow through the LED and recombine in the MQWs, emitting CP-EL (yellow helix). (b) Images of the LED off and on, showing the yellow EL. Reproduced with permission from ref 73. Copyright 2024, Springer Nature. (c) Schematic illustration of the chiral perovskite photodetector device architecture, showing the layer stack. (d) Wavelength-dependent photosensitivity (left *y*-axis) and photoconductor gain (right *y*-axis) of the chiral perovskite devices, comparing responses under LCP and RCP illumination for (S-α-PEA)­PbI_3_ and (R-α-PEA)­PbI_3_ enantiomers. Reproduced from ref 74. Available under CC-BY 4.0. Copyright 2019, Springer Nature.

### 2D Chiral Metal Halides

2D and quasi-2D (also regarded as Ruddlesden–Popper type) OIHPs are typically characterized by layered structures, in which inorganic octahedral sheets are connected through corner-sharing and are separated by layers of organic cations (can be chiral or achiral), which is unlike the chain-like configurations of 1D OIHPs, as shown in Figure 5 (b and c). This corner-sharing connectivity provides structural stability and facilitates efficient in-plane charge transport, while the bulky organic layers act as insulating barriers that confine carriers and excitons within the inorganic sheets. In some cases, edge-sharing connectivity can also be observed, although it is less common and often results in distorted lattice frameworks. One of the earliest reports on the 2D crystal structure of (R-/S-MBA)_2_PbI_4_ appeared in 2006,^29^ while its chiroptical properties were investigated later in 2017.^30^ Subsequently, in 2019, high-quality needle-shaped, millimeter-sized 2D single crystals were synthesized to investigate their CD and CPL properties. Interestingly, the CD measurement of (R-MBA)_2_PbI_4_ and (S-MBA)_2_PbI_4_ exhibited a mirror-type broad and single peak at 519 nm, which is different from the previous report.^30^ These materials also demonstrated average degrees of circularly polarized PL of 9.6% and 10.1% at 77 K, respectively, with a maximum degree of CPL reaching 17.6% for (S-MBA)_2_PbI_4_.^70^ Beyond their chiroptical responses, these chiral OIHP materials have shown great promise in spintronic applications, particularly in spin-LED devices, as shown in Figure 6. Spin-polarized current–voltage (I–V) measurements have demonstrated spin polarization levels as high as 86%, indicating their strong spin-filtering capabilities.^20^ This spin-selective behavior arises from the CISS effect, in which the chiral lattice preferentially transmits electrons of a particular spin orientation, resulting in spin-polarized currents even in the absence of magnetic materials. The coupling between the electron’s linear momentum and spin when traversing a chiral potential underlies this effect, enabling the unique spintronic functionalities observed in these materials.^12,16,20^ Figure 6 shows an LED device architecture in which 2D perovskite was used as a spin filter, demonstrating circularly polarized electroluminescence with degrees of polarization reported as 15  ±  4% and ±2.6%,^19,73^ depending on the device architecture and measurement conditions. This highlights the interplay among device design, chiral lattice properties, and spin-dependent charge transport. In addition, corner-sharing 2D tin-iodide perovskites were synthesized, exhibiting a significantly more distorted layered structure compared to previously reported tin-based analogues.^12^ These materials were systematically investigated for their chiroptical properties and spin-polarized charge transport characteristics.^12^ Remarkably, their CD spectra revealed multiple peaks in the 300–500 nm range, suggesting strong chiroptical activity. Even more remarkably, spin polarization extracted from current–voltage measurements reached values as high as 94%, underscoring the potential of these chiral perovskites in spintronic applications. Additionally, spin-polarized absorption and PL of OIHPs thin films were studied by controlling the dimensionality through chemically designed quasi-2D structures (consisting of two inorganic layers separated by organic spacers) via a combined process of chirality transfer and energy funneling.^50^ Interestingly, a 3% spin-polarized PL was observed even in the absence of an external magnetic field, attributed to the different emission rates of σ^+^ and σ^–^ polarized photoluminescence.

### 3D Chiral Metal Halides

3D OIHPs are characterized by a continuous framework of corner-sharing metal halide octahedra extending in all three crystallographic directions, stabilized by small organic cations occupying the interstitial voids, as shown in Figure 5d. Compared with their lower-dimensional counterparts, chiral 3D OIHPs exhibit reduced exciton binding energies and extended carrier diffusion lengths, making them particularly attractive for high-performance applications in chiral optoelectronics and spintronics. Despite their potential, the development of chiral 3D OIHPs remains challenging, predominantly due to the stringent requirement for compact organic cations that can fit within the 3D framework without distorting it or inducing a transition to lower-dimensional phases. However, the existence of chiral 3D OIHPs was initially supported only by theoretical predictions, particularly involving small chiral ligands such as (R-/S-CFMA)­PbI_3_ structures.^71^ These predicted structures are expected to be both kinetically and thermodynamically stable and to exhibit a range of multifunctional properties, including optical rotation, CD, SHG, piezoelectricity, pyroelectricity, and ferroelectricity, highlighting the urgent need for their experimental synthesis and comprehensive characterization toward next-generation multifunctional applications.^71^ Later, in 2021, homochiral single crystals of the 3D, particularly (R_3_HP)_2_RbBiBr_6_, were successfully synthesized (where R_3_HP = *R*3̅-hydroxypyrrolidinium).^72^ Interestingly, structural analysis of single crystals revealed that the compound adopts a unique mixed face- and corner-sharing connectivity pattern involving Rb^+^ and Bi^3+^ ions, forming a 4-connected ion topology characterized by a Schläfli symbol of 6^6^, which is relatively rare among hybrid perovskite frameworks. Owing to this distinctive crystal structure, the material also exhibits multifunctional chiroptical properties, including CD, CPL, SHG, ferroelectricity, and ferroelasticity. Very recently, a corner-sharing 3D octahedral network resembling that of traditional 3D perovskites was synthesized in chiral OIHPs of (R/S-3AQ)­Pb_2_Br_6_, which crystallizes in the typical orthorhombic *P*2_1_2_1_2_1_ chiral space group.^45^ This was achieved by carefully controlling the precursor ratio of the ditopic 3AQ and Pb­(OAc)_2_; otherwise, the reaction typically yields low-dimensional 2D structures.^45^ The chiroptical studies of this novel 3D structure revealed distinct CD signals in the range of 315–400 nm, with dissymmetry factors (*g*_abs_) on the order of 1 × 10^–5^, indicating strong chiral-optical coupling near the band edge and demonstrating promise for next-generation spintronic applications. However, to date, only two chiral 3D OIHP structures have been experimentally confirmed, with their chiroptical properties systematically investigated.^49^ This limited progress highlights the urgent need for further development in this field, particularly given the superior structural stability, extended charge transport pathways, and potential for intrinsic chirality offered by 3D architectures compared to their low-dimensional counterparts.

## Exploration of Chiral Metal Halide Films by Advanced X-ray Techniques

The analysis of complex chiral materials with the help of X-rays has become a central focus of materials research ever since the first groundbreaking success of resolving the structure of human DNA. In particular, chiral metal halides, whose asymmetric structures give them unique optical and electronic properties, are the focus of modern studies. Although single-crystal X-ray diffraction is a well-established method for determining crystal structures, resolving the structures of materials in the thin-film form remains challenging, particularly when the films are chiral and polycrystalline. Nevertheless, the first step typically involves performing X-ray diffraction (XRD) on the thin film and comparing the obtained pattern to the theoretical diffraction pattern of the corresponding single crystal. However, the characterization of chiral structures is not as straightforward in thin films as it is in single crystals. In this regard, the use of advanced X-ray scattering techniques, such as GISAXS (Grazing Incidence Small-Angle X-ray Scattering) and GIWAXS (Grazing Incidence Wide-Angle X-ray Scattering) has great potential. These methods offer unique insights into the hierarchy of material structures, ranging from nanoscale morphology to atomic lattice architecture (Figure 7a). A major advantage of these techniques lies in their ability to examine different depth ranges of a material by varying the angle of incidence. Below the material-specific critical angle of total external reflection, the interaction of the X-rays with the surface layer dominates, making the surface morphology visible. Above the critical angle, on the other hand, the scattering depth is increased so that information can be obtained from deeper layers right down to the bulk. This ability to determine the structure complementarily from the surface to the interior of the material is particularly important for thin films and layered materials, whose properties are often characterized by the interaction between the surface and bulk. The combined application of GISAXS and GIWAXS offers unique opportunities to capture the structure of a material on a hierarchical level from nanometer to Ångström: On the one hand, GISAXS is ideally suited to study nanoscale features such as surface and bulk morphology and its organization in subdomains of lateral structures and along the vertical sample profile (Figure 7b). In studies on chiral metal halide films, GISAXS showed how the spin coating time influences the size distribution of the obtained crystals and extends the morphological knowledge from surface-limited electron microscopy to the bulk interior of the films.^77^ On the other hand, GIWAXS is specifically designed to provide crystallographic information on the lattice structure as a function of momentum transfer *q*(Å^–1^). A unique feature of GIWAXS is its ability to capture the in-plane orientations of crystallites in thin films as a function of the azimuthal angle χ (°) (Figure 7c). This information is not accessible from conventional XRD, which primarily measures orientation along the surface normal (χ = 0°), as indicated by the red dashed line in Figure 7c.^20^ By combining GIWAXS and XRD, researchers obtain a complete picture of the three-dimensional crystal orientation in thin films, as XRD complements the missing wedge in GIWAXS. In studies on chiral metal halides, for example, GIWAXS was able to show that solvent modulation leads to in-plane distortions and a varying orientation of crystallites.^78^ By varying the angle of incidence, the researchers were also able to reveal the depth dependence of the crystal phase. This in-plane structural information is essential in understanding the relationship between the atomic lattice architecture and optical properties such as circular dichroism. Furthermore, GIWAXS revealed the influence of solvents and deposition techniques on the amount of chiral perovskite crystallites in a certain orientation.^10,79^ In combination, GISAXS and GIWAXS techniques enable comprehensive characterization of the hierarchical structure of materials in-plane and out-of-plane, as well as from surface to bulk. This ability to reveal structure–function relationships at multiple levels is key to the development of new materials with tailored properties. Moreover, with the high brilliance at synchrotron facilities and the capability to mount custom-made experimental environments, advanced insights in the field of chiral metal halides are obtained via in situ and operando experiments of high temporal and spatial resolution, which holds promise for a bright future in the nanoscience community. Despite some success, determining the crystal structures of chiral metal halides by using these scattering techniques presents several challenges. First, the inherent polycrystallinity of thin films and the uncontrollable orientation of grains often result in complex diffraction patterns with overlapping scattering peaks, making it difficult to extract structural information. Second, chiral metal halide materials crystallize in non-centrosymmetric space groups, which are more challenging to resolve due to lower symmetry. Furthermore, distinguishing between enantiomers solely based on these techniques is extremely difficult due to the lack of sensitivity of standard X-ray scattering methods to the absolute configuration of materials. Therefore, an in-depth understanding of the diffraction patterns of chiral metal halides is essential for accurately extracting their crystal structures and determining their handedness in the thin-film form.

**Figure 7:**
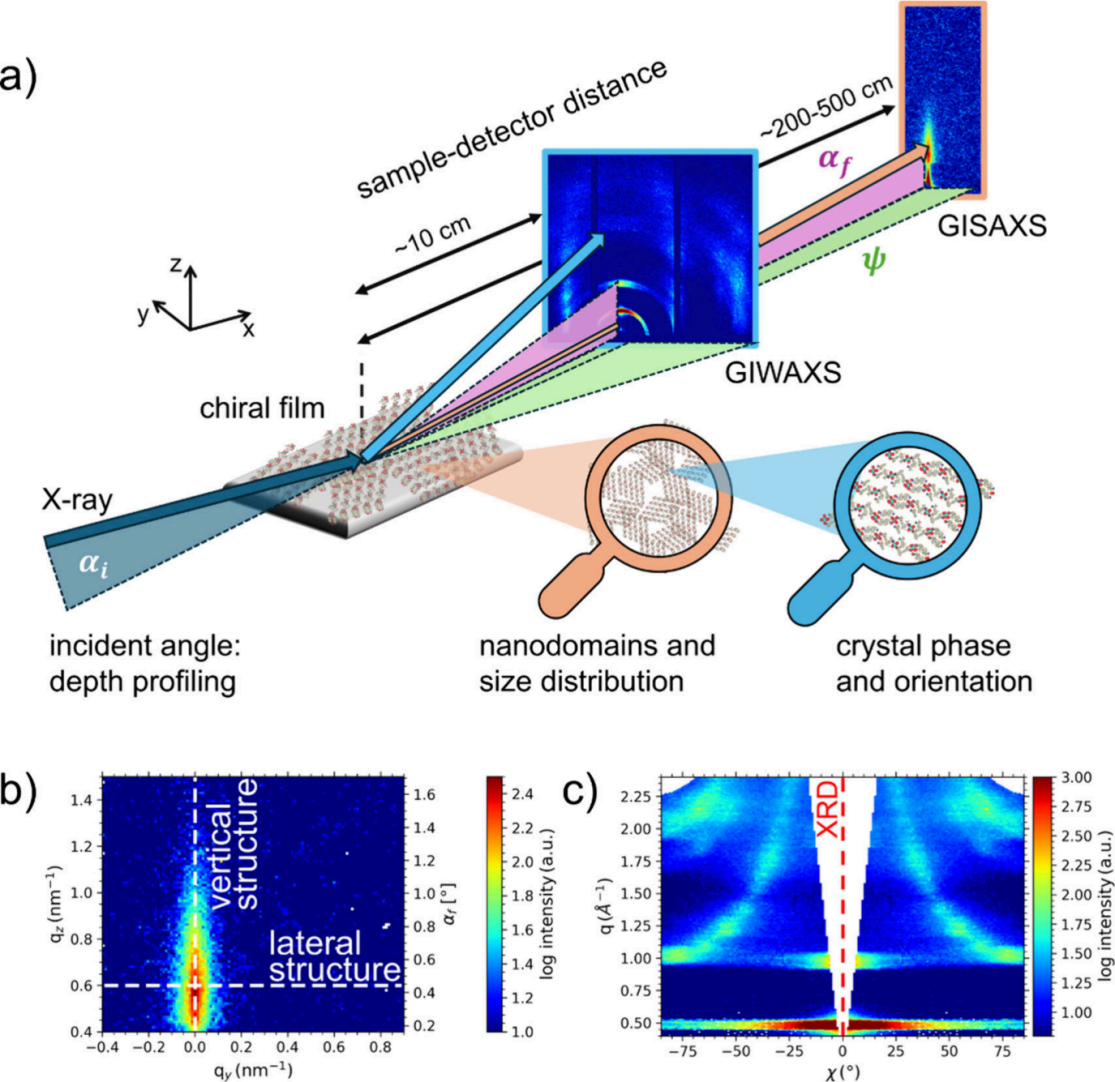
(a) Schematic of a GISAXS/GIWAXS experiment. (b) 2D GISAXS data, from which vertical and lateral information on the nanostructure is obtained. (c) Reshaped 2D GIWAXS data in sector plot representation, revealing the lattice structure and its orientation of chiral perovskites with respect to the substrate. The missing wedge of inaccessible data is complemented by XRD.

## Colloidal Chiral Perovskite Nanocrystals

Although chiral metal halide bulk materials exhibit strong chiroptical responses, their weak emission has shifted the focus of circularly polarized luminescence (CPL) generation toward colloidal nanocrystals.

Although obtaining chiral 3D perovskite nanocrystals (NCs) remains challenging, template induced self-assembly and cholesteric liquid crystal filtering have shown great promise for generating circularly polarized luminescence (CPL) from achiral NCs.

Colloidal chiral perovskite nanocrystals have recently emerged as a promising class of CPL emitters owing to their high PLQY and tunable emission across the visible spectrum by halide composition and through accessing quantum confinement properties (Figure 8a).^80–82^ Currently, various groups are exploring different methods to induce chirality in perovskite NCs. In general, there are three common approaches to achieving chirality in colloidal NCs: 1) the use of chiral ligands either in direct synthesis via reprecipitation or by postsynthetic ligand exchange (Figure 8b and c), 2) chiral template-assisted assembly into chiral structures, as illustrated in Figure 8d, and 3) shaping NCs into twisted or helical morphology, a widely applied method to chiral plasmonic NCs, but not yet been successfully explored for perovskite NCs.^37^ In direct synthesis, chiral ligands alone cannot stabilize the NCs, as they are often small molecules with weak binding affinity and insufficient steric stabilization. Therefore, the synthesis of chiral NCs is often carried out in the presence of a mixture of chiral molecules (Typically, (R/S)-phenylethylamine (PEA), (R/S)-methylbenzylamine (MBA), (R/S)-α,4-dimethylbenzylammonium (DMBA), etc., in the form of ammonium halide) and long-chain amines such as octylamine and oleylamine, which provides additional steric stabilization and surface passivation. Different chiral ligands used in the literature, along with the g-factors achieved for various perovskite systems, are summarized in Table 2. The reprecipitation approach at room temperature (or relatively low temperatures) generally yields quantum-confined perovskite nanoplatelets (NPLs). The CD signals of NPLs increase with increasing the concentration of chiral ligand relative to achiral ligand, but with excess concentration, the NPLs tend to aggregate and lose their chirality.^83^ The choice of achiral ligands should be based on their binding affinity to the perovskite NC surface to ensure effective stabilization.^84,85^ The NCs with opposite handedness exhibit mirror-image-like CD spectra with opposite signs. The reported g-factors (g_lum_ - luminescence anisotropy and g_CD_ -absorption anisotropy) for chiral colloidal NCs typically fall within the range of 10^–2^ to 10^–5^.^86^ It is worth mentioning that g_lum_ and g_CD_ can be slightly different for the same chiral NCs. A study has shown that the magnitudes of g_lum_ and g_CD_ exhibit linear correlation with g_lum_ = 0.4 × g_CD_.^83^

**Figure 8:**
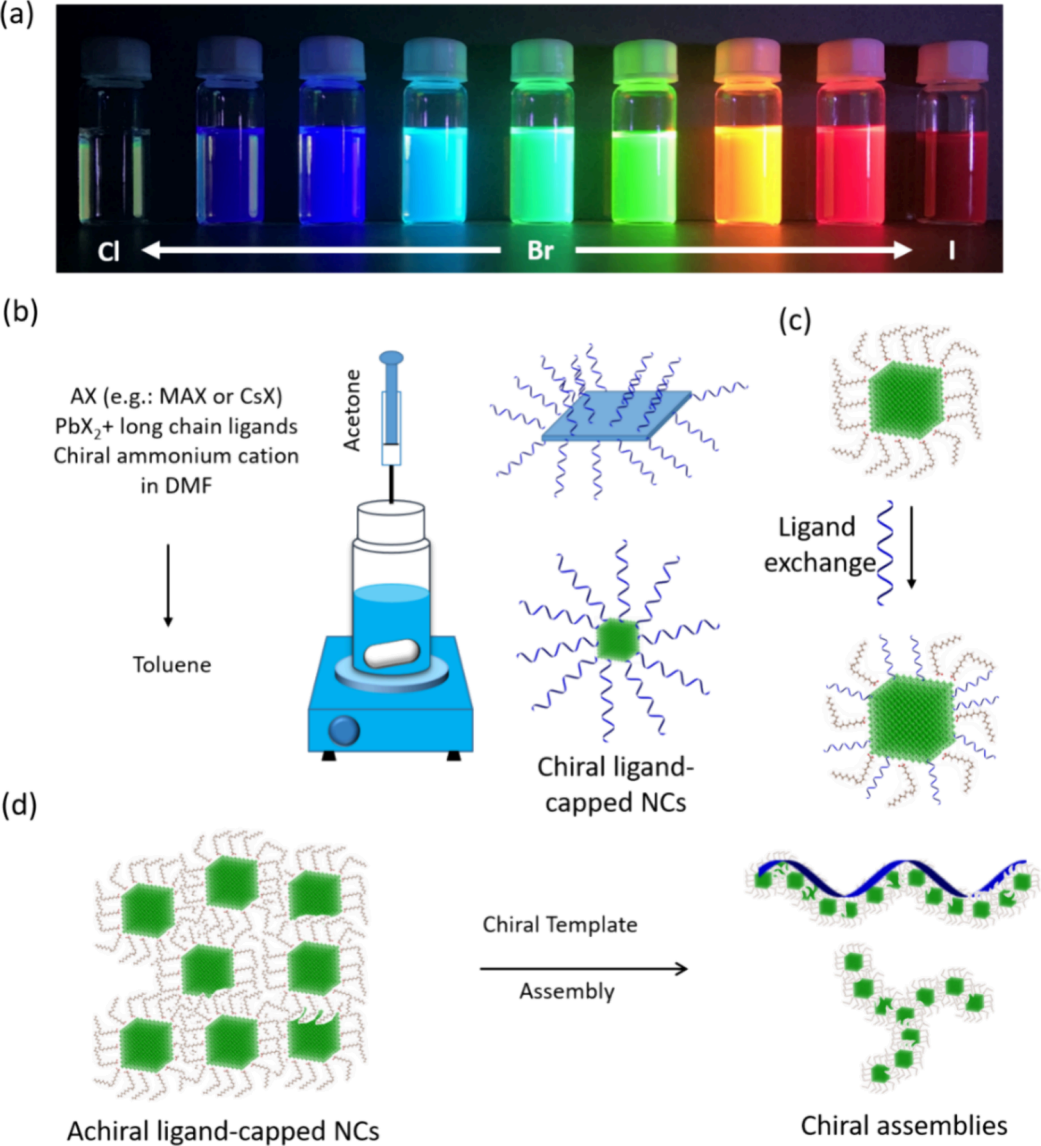
(a) Photograph of colloidal solutions of chiral perovskite nanocrystals. (b) Schematic illustration of the typical synthesis of chiral perovskite nanocrystals (mixture of chiral and achiral ligand-capped NCs) by the reprecipitation approach. This approach often yields quantum-confined nanoplatelets or quantum dots. (c) Inducing chirality in NCs by postsynthetic exchange of an achiral ligand with a chiral ligand. This is an effective approach for obtaining quantum-confined chiral nanocrystals. Although this method has also been applied to nanocubes, achieving chiral 3D perovskite NCs is still challenging. (d) Schematic illustration of chiral template-assisted assembly into chiral architectures (e.g., helical, triskela, gammadions, etc.) that emit CPL. The template can be part of the assembly, or it can be removed after the formation of assemblies.

**Table 2:** Summary of Various Chiral Ligands Used to Obtain Different Types of Chiral Metal Halide Perovskite NCs and Their Dissymmetry Factors (g_CD_ and g_lum_), Along with Excitonic Peak Wavelength and CD Signal Intensities^a^

No	Ligand: NCs	CD signal intensity at excitonic peak (mdeg)	Excitonic peak, Absorption range (nm)	g_cd_	g_lum_	Ref
1	(R/S)-α-octylamine: CsPbBr_3_	4	500 (400–500)	2.3 × 10^–4^	525 (7 × 10^–3^)	^96^
2	(R/S)-1-phenylethylamine: CsPbBr_3_	30	428 (350–450)	2 × 10^–3^	--	^94^
3	(R/S)-1-(1-naphthyl)ethylamine: CsPbBr_3_	4	435 (350–450)	--	--	^94^
4	(R/S)-2-aminooctane: CsPbBr_3_	20	430 (400–450)	3.5 × 10^–3^	--	^94^
5	(R/S)-β-methylphenethylammonium bromide: CsPbBr_3_	15	440	15 × 10^–3^	--	^88^
6	(L/D)-tryptophan: CsPbBr_3_	–20	500	--	500 (2.3 × 10^–3^)	^111^
7	(R/S)-methylbenzylammonium bromide: CsPbBr_3_	15	460 (300–500)	2.9 × 10^–4^	470 (5.2 × 10^–4^)	^89^
8	Chiral cyclohexylethylamine, CHEA: CsPbBr_3_	13	508	2.5 × 10^–3^	515 (2.7 × 10^–4^)	^91^
9	(R/S)- serine carbon dot: CsPbBr_3_	1	300–500	1.5 × 10^–3^	520 (−3.1 × 10^–3^)	^112^
10	(R/S)-2-aminooctane (2-AO): CsPbBr_3_	22	445 (300–500)	--	--	^113^
	(R/S)-Methylbenzylamine (MBA): CsPbBr_3_	20	445 (300–500)			^113^
11	enantiomeric 1,2-diaminocyclohexane:[CsPb(I/Br)_3_]	240	280	1.5 × 10^–3^	400, 440	^114^
12	(R/S)-phenylethylammonium bromide: CsPbBr_3_ NPLs	2	425 (400–460)	1 × 10^–5^	--	^93^
13	(L/D)-arginine: Cs_3_Cu_2_Br_5_	4	280, 250 (225–350)	1.6 × 10^–4^	--	^115^
14	(R/S)-2-aminooctane:MAPbBr_3_	1.5	445 (300–500)	1.5 × 10^–4^	--	^116^
	(R/S)-1-(1-naphthyl)ethylamine: MAPbBr_3_	1	445 (300–500)	0.6 × 10^–4^	--	^116^
15	(*R*)-(+)-α,4-dimethylbenzylammonium: MAPbBr_3_	300	405 (400–460)	8 × 10^–4^	425 (2.3 × 10^–3^)	^83^
16	(R/S)-Methylbenzylamine (MBA): FAPbBr_3_	--	--	--	510 (1.18 × 10^–2^)	^95^
	(R/S)-2-aminooctane (2-AO): FAPbBr_3_	--	500	7 × 10^–2^	526 (2 × 10^–2^)	^95^
17	Chiral Liquid crystal-MA/CsPb(Cl/Br/I)_3_	--	(450–625)		Up to 1.9	^102,109,108,37^
18	CsPbBr_3_ and CsPbI_3_ NCs metasurfaces		500, 650		Up to 1.3	^85^
19	chiral gelator-CsPb(Cl/Br/I)_3_ NCs		(420–600)		Up to 10^–3^	^105^
20	Chiral metasurfaces-induced CPL from CsPbX_3_ NCs		500–650		Up to 0.5	^107^

a MA: Methylammonium, FA: Formamidinium, CHEA: Cyclohexylethylamine, CD: Circular dichroism, CPL: Circularly polarized luminescence, NCs: Nanocrystals.

Unlike bulk chiral metal halides, in which the chiral molecules are incorporated into the crystal lattice in most cases, colloidal NCs possess chiral ligands only on their surfaces. Thus, the efficiency of the chiroptical activity (g-factor) of NCs strongly depends on their surface area and the density of chiral ligands.^37,83,87–91^ As the surface area increases with a decrease in the dimensions (size or thickness) of the NCs, their chiroptical activity correspondingly increases.^50,87,88,91^ Therefore, chiral ligand-capped NCs or NPLs with strong quantum confinement exhibit higher g-factors compared to their bulk counterparts.^87^ The CD spectra of the chiral NCs/NPLs often exhibit the Cotton effect, characterized by the splitting of the CD peak at the excitonic position into positive and negative signals.^85^ Although the mechanism of chirality transfer from ligands to NCs remains unclear, it is generally believed that chiral ligand-induced surface distortion, rather than a hybridization of electronic states of ligands and perovskite NCs, is primarily responsible.^87,92^ The surface distortion leads to symmetry breaking in the perovskite lattice and spin-splitting of the conduction bands and is very effective in strongly quantum-confined NPLs compared to 3D NCs.^87^ However, it remains challenging to achieve precise control over the thickness of chiral perovskite NPLs. The reprecipitation reactions often yield NPLs with mixed thicknesses, resulting in overlapping their CD signals. Moreover, long-term stability of chiral NPLs remains challenging, and there is a need for the development of chiral ligands that can strongly bind to the NC’s surface and preserve their chiroptical properties over time by preventing aggregation. To obtain chiral NCs with desired morphology, postsynthetic ligand exchange of presynthesized achiral NCs with a chiral molecule can be performed, and this approach has been successfully applied to quantum-confined and 3D perovskite NCs.^88,93–95^ It was found that such postsynthetic chiral ligand exchange could result in inversion of the intrinsic chirality of colloidal NPLs that arises from lattice dislocations. The ligand exchange strategy has also been applied to understand the size effects on ligand-induced chirality, and it has been found that the chiroptical activity decreases with increasing size.^88^ Although a few studies have shown chiral ligand-induced CD response from 3D colloidal NCs,^95,96^ it remains extremely challenging to obtain colloidal 3D chiral perovskite NCs without confinement or weak confinement. The ligand exchange strategy faces a major hurdle, which is the insolubility of chiral ligands (amine bromide salts) in organic solvents. To overcome this, chiral ligands were first dissolved in polar organic solvents such as DMF and ethyl acetate by sonication, and then were used to treat perovskite NCs dispersed in hexane or toluene.^93,95^ The chiral ligands, such as R/S-methylbenzylamine, R/S-1-naphthylethylamine, and R/S-methylphenethylamine (in the form of ammonium halides), have been typically used for ligand exchange on NPLs or nanocubes.^88,93–95,97^ It was demonstrated that the ligand exchange method can produce chiral FAPbBr_3_ NCs with an average *g*_lum_ = ±1.18 × 10^–2^.^95^ The origin of chirality in such weakly confined perovskite NCs is attributed to chiral ligand-induced structural distortions in the crystal lattice.^92^ However, a few studies have shown that weakly confined NCs exhibit very weak CD signals.^88^ Consequently, further investigation of chiral NCs of varying sizes is needed to better understand the origin of chirality. Nevertheless, the g-factors (10^–2^ to 10^–5^) of chiral ligand-capped NCs remain relatively low compared to those observed in other nanoparticle systems, such as gold. Chiral gold NCs have been shown to exhibit ensemble g-factors of up to 0.2.^98,99^ However, their chirality is intrinsic, arising from their twisted or helical morphology. A few studies have reported intrinsic chirality in perovskite NCs with g-factors in the range of 10^–2^.^44,100,101^ In these systems, the chirality was attributed to the orientation of nanocubes connected into 1D nanowires^100^ or foreign metal ion-induced spiral distortion of the lattice along the axial direction of ultrathin nanowires.^101^ Significant opportunities remain to enhance the g-factors of intrinsically chiral perovskite NCs by shaping them into twisted or helical morphologies. The major challenge in this regard lies in slowing down nucleation and growth to achieve better control over morphology.

Another important strategy for achieving chiral emission from perovskite NCs involves assembling them into chiral architectures using nanoscale or microscale templates. The chiral templates, such as helical polymer nanofibers,^103^ inorganic silica nanohelices,^104^ and chiral gelators,^105^ have been used to assemble perovskite NCs into helical assemblies that emit CPL with g_lum_ in the range of 10^–2^-10^–3^. These chiral nanotemplates typically possess functional groups capable of binding to perovskite nanocrystals, thereby facilitating their self-assembly into a template-defined morphology. However, these nanotemplates often result in irregular chiral assemblies with relatively low g-factors. Alternatively, PDMS template-assisted nanoimprint lithography has been employed for creating a large area and uniform 2D chiral perovskite NC patterns (metasurfaces) with a g_lum_ up to 0.3 (Figure 9a).^106^ In addition, the chiral metasurfaces made with high refractive index materials such as TiO_2_ can be used to induce CPL from achiral perovskite NCs with a g_lum_ up to 0.5.^107^ The CPL can be tuned across the visible spectrum by varying both the halide composition of the perovskite nanocrystals and the design of the chiral metasurface, whose CD response is matched to the absorption of the nanocrystals.^107^ Another promising approach for achieving chiral emission from perovskite NCs is to combine them with chiral liquid crystals (CLCs) in a layer-by-layer or blended form (Figure 9b).^37,108,109^ The CLCs are photonic materials that selectively reflect light with one specific polarization (L or R), depending on their chirality. The R-CLC reflects R-polarized light and vice versa. Thus, one can achieve CPL from perovskite NCs across the visible range by varying the composition and photonic bandgap of CLCs.^108^ This method could generate CPL from perovskite and other NC systems with g_lum_ up to 1.9 (Figure 9b).^37,102^ However, this approach results in significant energy loss, as it relies on the selective reflection of light. Despite significant progress in achieving chiral emission from perovskite NCs, a few attempts have been made toward the fabrication of LEDs that emit CP electroluminescence with g_CPEL_ in the range of 10^–3^ using chiral perovskite NC emitters (Figure 9c).^17,110^ Therefore, the fabrication of chiral LEDs remains a critical area of research to advance g_CPEL_ and EQE to the levels required for practical, real-world applications.

**Figure 9:**
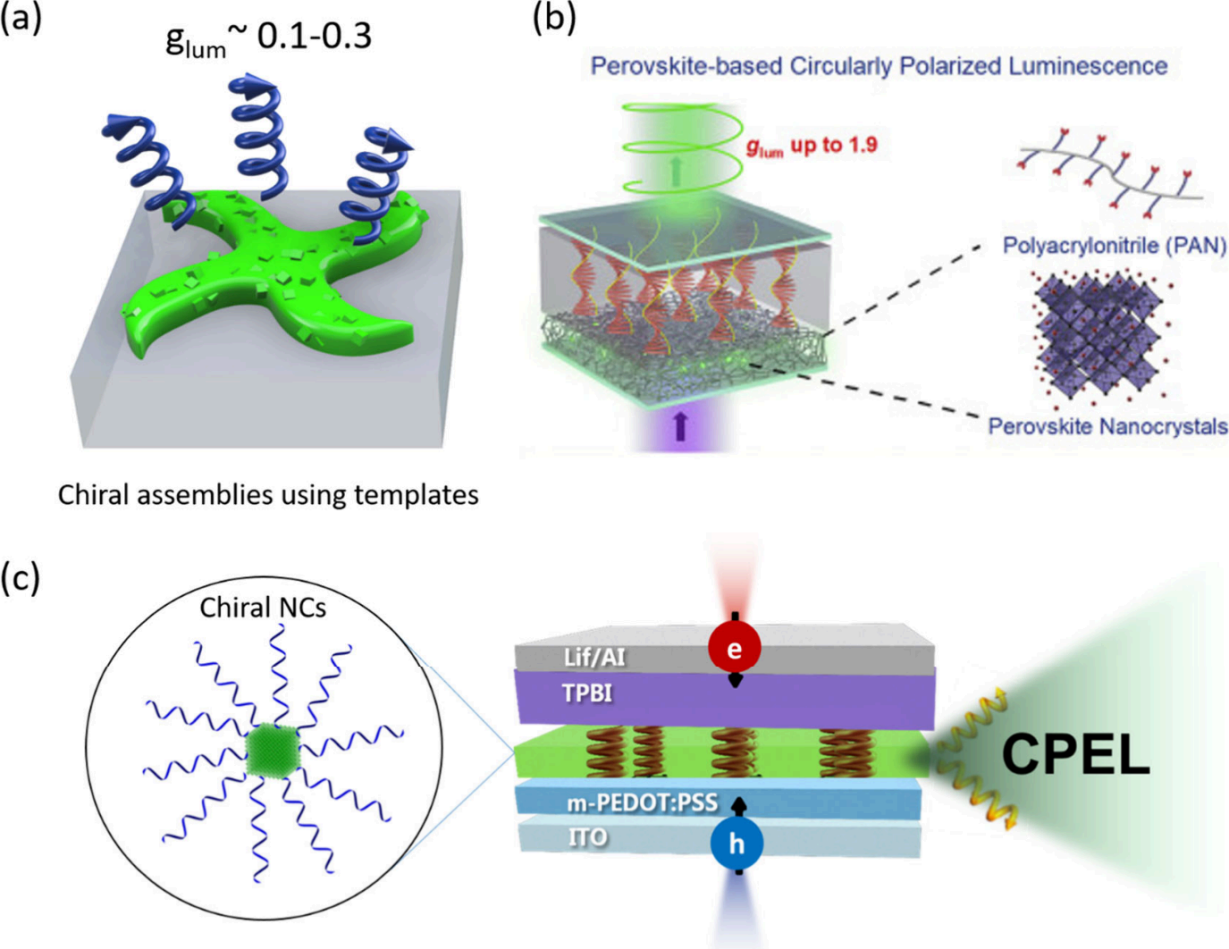
(a) PDMS template-assisted chiral architectures (triskelion morphology) emitting CPL with g_lum_ ∼ 0.1–0.3. (b) Achieving chiral emission from perovskite NCs by using chiral liquid crystal filters. Reproduced from ref 102. Copyright 2022, with permission from Elsevier. (c) A typical LED device architecture for generating circularly polarized electroluminescence (CPEL) using chiral perovskite NCs emitters.

In summary, this Perspective discusses the advances and outstanding challenges of chiral perovskite and perovskite-inspired bulk single crystals and colloidal nanocrystals, focusing on their design and synthesis, as well as the relationship between structure and chiroptical properties. The incorporated chiral organic cations not only dictate the crystal dimensionality, ranging from 0D to 3D frameworks, but also strongly influence crystal symmetry, chiroptical handedness, and electronic structure. The introduction of chiral ligands into the crystal lattice or on the surface of NCs leads to distinctive optical phenomena, including strong CD and CPL, which hold great promise for advancing next-generation spintronic and optoelectronic applications. The CD response is tunable by the composition of metal and halide ions and their dimensionality. Different combinations of chiral ligands, metal (Pb and Pb-free), and halides have been explored in the preparation of chiral metal halides to improve their g-factor through a fundamental understanding of structure/composition-property relationships. Studies have shown that CD signals are enhanced as the dimensionality decreases from 3D to 2D. They have already shown great success in the fabrication of chiral LEDs, CP light detectors, and SHG. In particular, they showed significant promise for selective electron spin filtering via the CISS effect, and this concept has already been demonstrated in the fabrication of circularly polarized (CP) LEDs. The metal ion, the size of the chiral molecules, and their functional groups, typically ammonium halides, are crucial factors in the design of chiral metal halides with desired dimensionality. On the other hand, chiral colloidal NCs have been receiving increased attention owing to their facile synthesis and self-assembly behavior and the resulting chiroptical properties. Similar to bulk systems, the g-factors increase with decreasing size of colloidal NCs. A wide range of chiral colloidal NCs, spanning from lead-based to lead-free compositions and from hybrid to all-inorganic perovskites, have been reported. Chirality in colloidal NCs can be achieved through the use of chiral ligands in direct synthesis or postsynthetic ligand exchange or by chiral template-assisted self-assembly. The g-factors of chiral bulk crystals typically range from 10^–4^ to 10^–2^, whereas those of colloidal NCs and their assemblies span a broader range, from 10^–3^ up to 0.5. Despite significant progress in bulk single crystals and NCs of chiral OIHPs over the last 5 years, numerous challenges and opportunities remain to be addressed to realize their full potential in optoelectronic devices.

Currently, precise control over the dimensions of bulk chiral crystals remains limited. Existing methods, primarily the TLM approach or autoclave-assisted synthesis, offer restricted tunability and scalability, highlighting the need for a more versatile method for the preparation of large-scale, environmentally sustainable, and stable chiral perovskites. In the case of thin films, controlling the orientation of chiral perovskites with respect to the substrate is crucial to harnessing their maximum efficiency in corresponding optoelectronic devices. Achieving this requires a deeper understanding of chiral-symmetry-dependent GIWAXS and GISAXS patterns. The crystallographic symmetry of chiral OIHPs plays a crucial role in controlling their energy-relevant chiroptical and spin-selective behaviors. Most chiral OIHPs crystallize in typical chiral space groups (highlighted in gray in Figure 2a), such as *P2*_*1*_ *2*_*1*_ *2*_*1*_. In contrast, a few chiral metal halide compositions crystallize in helical space group pairs (e.g., *P*4_3_2_1_2 and *P*4_1_2_1_2; P6_1_ and P6_5_) that produce mirror-image crystal structures with opposite chiroptical responses. There are still several helical space group pairs that have not yet been reported for metal halide systems. Moreover, a clear understanding of the structure–property relationship is still lacking in chiral perovskite and perovskite-inspired metal halides. A systematic investigation of g-factors and the CISS effect of different compositions and crystal structures is needed under similar experimental conditions. To date, chirality in these systems is attributed to the asymmetric interactions between chiral molecules and inorganic metal halides, leading to dissymmetry in their electronic transitions. However, the role of lattice helicity or anisotropy on the chiroptical properties is not clear. 3D chiral OIHPs offer significant advantages, including enhanced structural stability, lower exciton binding energies, and longer carrier diffusion lengths, making them highly promising candidates for high-performance optoelectronic and spintronic devices. Despite these benefits, their development remains significantly underexplored compared to that of their lower-dimensional (2D, 1D, and 0D) counterparts. This is largely due to the stringent requirements for compact chiral organic cations that can fit within the 3D perovskite framework without inducing dimensional reduction or structural distortion. This limited progress highlights an urgent requirement for innovative molecular design and synthetic strategies to explore the full potential of chiral 3D perovskites in scalable, next-generation technologies. We strongly believe that training an artificial intelligence (AI) model based on the existing literature database could be useful for the design of new chiral metal halides with desired crystal structures and chiroptical properties.

As for colloidal chiral NCs, stability and composition tunability are the major challenges. The chirality in colloidal NCs is typically imparted by the chiral ligands on their surfaces. However, due to their weak binding, chiral ligands tend to desorb from the surface of NCs and render the NCs optically inactive within a few hours after the synthesis. Moreover, to date, most studies have been focused on Br-based perovskites, but there is significant room to expand the studies to other compositions. The chiroptical activity of colloidal NCs strongly depends on their dimensions, as the CD response tends to be diminished with increasing NC size. Despite a few reports, it remains challenging to obtain 3D colloidal chiral perovskite NCs. On the other hand, template-assisted chiral assemblies with relatively high g-factors show great promise for optoelectronics, but their implementation into devices remains challenging. We anticipate that the combined use of bulk perovskite films as spin filters and chiral colloidal NCs as emissive materials in a layer-by-layer configuration can enable chiral LEDs exhibiting high polarization anisotropy.
